# *Staphylococcus aureus* carriage and prevalence of skin and soft tissue infections among people who inject drugs: a longitudinal study

**DOI:** 10.1038/s41598-024-63574-y

**Published:** 2024-06-05

**Authors:** Jimmy Jörgensen, Disa Dahlman, Marianne Alanko Blomé, Håkan Janson, Kristian Riesbeck, Anna C. Nilsson

**Affiliations:** 1 Clinical Infection Medicine, Department of Translational Medicine, Faculty of Medicine, Lund University, Malmö, Sweden; 2 Department of Clinical Sciences Center for Primary Health Care Research, Malmö, Faculty of Medicine, Lund University, Malmö, Sweden; 3 Division of Psychiatry, Department of Clinical Sciences Lund, Faculty of Medicine, Lund University, Lund, Sweden; 4 Clinical Microbiology, Central Hospital, Växjö, Sweden; 5 Clinical Microbiology, Department of Translational Medicine, Faculty of Medicine, Lund University, Malmö, Sweden

**Keywords:** Infectious diseases, Clinical microbiology

## Abstract

People who inject drugs are frequently colonized with *Staphylococcus aureus* and have an increased risk for skin and soft tissue infections. This longitudinal study aims to describe *S. aureus* carriage in this group and the risk for infections during a 1-year follow-up. We included 61 participants from the Malmö Needle Exchange Program. Mapping of *S. aureus* carriage was conducted by screening cultures every third month and *S. aureus* growth was semi-quantified. Data regarding infections and living conditions were collected from structured interviews. Statistics included univariate analysis with the Fischer’s exact test, univariate logistic regression and multivariate logistic regression. *S. aureus* carriage was detected in 46–63% of participants, and 75% reported one or more infections during the study period. Self-reported infections were associated with carriage in perineum (OR 5.08 [95% CI 1.45–17.73]), in skin lesions (OR 1.48 [95% CI 1.21–1.81]), and unstable housing situation (OR 12.83 [95% CI 1.56–105.81]). Thus, people who inject drugs are frequent carriers of *S. aureus* and report a surprisingly high prevalence of skin and soft tissue infections. Homeless people and those with skin carriage seem to be at highest risk. Effective clinical interventions are needed, aiming at preventing infections in this vulnerable group.

## Introduction

*Staphylococcus aureus* is a human commensal, but also a common cause of both community acquired and nosocomial infections. People who inject drugs (PWID) have an increased risk for both skin and soft tissue infections (SSTIs) and more severe systemic infections, such as septicaemia, endocarditis and spondylodiscitis^1,2^. In this context, *S. aureus* is the most common causative agent^2–4^. In international studies self-reported lifetime SSTI prevalence varies from 46 to 68% in the PWID group^5–9^, and in a Swedish study performed in our area and setting (Malmö Needle Exchange Program; NEP), the self-reported lifetime SSTI prevalence was 58%^10^. Compared to the average population, PWID are more frequent carriers of *S. aureus*^11–13^, and it has also been shown that *S. aureus* carriage is a risk factor for development of subsequent infections^14,15^. PWID also have other predisposing risk factors for infections, such as inadequate skin cleansing and hand washing before injection^2,16^.

The aim of this study was to describe the prevalence and longitudinal pattern of *S. aureus* carriage among PWID, and to explore its association with the incidence of self-reported SSTIs. To date, there has been limited research on *S. aureus* bacterial carriage and SSTIs among PWID in longitudinal settings.

## Materials and methods

### Setting

The Malmö NEP, where this prospective longitudinal observational cohort study was conducted, is a part of the Department of Infectious Diseases at Skåne University Hospital in Malmö. The NEP is located in a separate building, with its own staff ensuring privacy. The Malmö NEP provides participants with sterile injection equipment, such as needles, syringes, filters and cups, regarding their individual needs. Mixed use of drugs is common and approximately 30% of the participants are women. Surveillance for bloodborne viruses is conducted, and the participants are offered both treatment and vaccination when possible. Outpatient healthcare including care of wounds and incision of abscesses is offered on site together with provision of full courses of antibiotics. All services are free of charge. The Malmö NEP is located in Skåne region in southern Sweden, in the Malmö metropolitan area, which has an approximate population of 355,000 inhabitants. During the study period a total of 1200 individuals with injection drug use were estimated to live in Skåne region, of whom approximately 80% used one of the four existing needle exchange services in the region. The Malmö NEP had 600 active annual participants, accounting for around 8000 visits^17^.

### Study participants

All participants in the Malmö NEP were invited by the NEP personnel to participate in the study from December 2016 to September 2017. Written informed consent was obtained from all study participants. Exclusion criteria were language difficulties and psychiatric illness, serious enough to aggravate study completion. Invitations were made by the NEP personnel considering the exclusion criteria, but also based on personal knowledge regarding expected compliance. After sampling procedures, participants were offered a small gift, mainly hygiene related products. The study was approved by the Regional Ethical Review Board in Lund (file number 2015/59), and all research has been performed in accordance with relevant guidelines and regulations.

### Data collection

During one year after inclusion of each participant, structured interviews were performed by the ordinary staff at every Malmö NEP visit, however, not more frequent than every two weeks. The interviews were based on a questionnaire evaluating clinical infections, access to shower and housing situation since the last interview. Self-reported SSTIs were not confirmed with clinical examination or bacterial cultures.

### Sampling

Mapping of *S. aureus* carriage was conducted by repeated cultures every third month. Samples were collected from the participant’s anterior nares, throat, perineum and skin lesions. Samples from existing skin lesions were collected, regardless of any signs of infection. Separate swabs were used at the different body sites. Samples from the anterior nares, throat and skin were collected by the NEP personnel. The perineal sampling was conducted by the participants themselves in privacy, after instructions. The first swab was inserted 1 cm in each nostril and rotated thrice, the second one was streaked twice on one of the tonsils and the third one was streaked thrice on the perineum. After cleansing skin lesions, samples were obtained from the edges. The swabs were stirred for 30 s in Copan E-swab transport medium (480CE; Copan Italia, Brescia, Italy) in separate tubes, before they were transported to the microbiological laboratory.

### Culture conditions

Semi-quantitative *S. aureus* cultures were done by adding 100 µl of each sample to BBL CHROMagar Staph aureus plate (CHROMagar, Paris, France). The inoculum was spread on the plates with sterile glass beads and incubated at 35 °C. After 48 h, colonies with typical colour appearance were confirmed by agglutination (Pastorex Staph Plus [Bio-Rad, Hercules, CA]). In case of growth of colonies with atypical colour, Pastorex agglutination and MALDI-TOF (Microflex LT, Bruker, Bremen, Germany) were used to determine atypical *S. aureus*. Due to laboratory problems, 0.3% of the collected cultures could not be analysed. The number of viable bacteria was measured as colony forming units (CFUs). Semi-quantitative evaluation of *S. aureus* growth was made by counting the CFUs, where CFUs ≥ 500 were considered as abundant, 50–499 as intermediate, 1–49 as sparse and 0 as no growth. Semi-quantification was mistakenly not performed in 3.8% of the *S. aureus* findings in this study.

### Analysis

Questionnaire data and microbiological data are reported descriptively. During each three-month period of the study, we documented the number of individuals who reported one or more infections and the number of individuals who had *S. aureus* growth verified through culture in various body sites: (a) anterior nares, throat, perineum and skin lesions; (b) any body site; and (c) two or more body sites simultaneously. During analysis, we took into account the consecutive inclusion manner, and distinguished the study periods for each patient.

In addition, we analysed associations between the outcome variable self-reported SSTI and covariates (gender, age, housing situation, shower access) and culture verified *S. aureus* carriage. Univariate analysis by Fischer’s exact test was used for all factors except from age, where univariate logistic regression analysis was used. For multivariate logistic regression analysis, the covariates age, gender, housing situation and *S. aureus* in perineum were selected. All analyses were performed using IBM SPSS Statistics 26^18^. A *p* value < 0.05 was considered as statistically significant.

## Results

### Sample characteristics

Sixty-one individuals (57% men, mean age 42 years) were included in the study (Table 1). More than one-third reported unstable housing, and one-sixth reported unstable shower access at any time point during the study period. Besides from SSTIs, other infections were commonly reported during the study (128 cases in 46 individuals). Most frequent was upper airway infections (*n* = 88), dental infections (*n* = 16) and urinary tract infections (*n* = 8). Reported number of hospital admissions was 29, but only in 5 cases, the probable cause was an injection related infection (Table 2).

**Table 1:** Baseline characteristics of study subjects (*n* = 61).

Characteristics	
Sex, *n* (%)	
Male	35 (57)
Female	26 (43)
Mean age (years ± SD) at inclusion (range)	41.9 ± 12.2 (18–65)
Housing situation last 15 months, *n* (%)	
Stable	38 (62)
Unstable	23 (38)
Shower access last 15 months, *n* (%)	
Stable	51 (84)
Unstable	10 (16)

**Table 2:** *Staphylococcus aureus* culture findings and self-reported infections (% [*n*/*N* of sampled individuals]) during the defined time periods.

Time period (month)	1–3	4–6	7–9	10–12	13–15
Number of cultures per individual, median (range)	1 (1–2)	1 (0–1)	1 (0–1)	1 (0–2)	1 (0–1)
Number of questionnaires per individual, median (range)	3 (1–7)	4 (0–6)	3 (0–6)	2 (0–6)	2 (0–7)
Bacterial findings					
* S. aureus* in nares, % (*n*/*N*)	39 (24/61)	37 (17/46)	37 (18/49)	29 (12/41)	31 (11/35)
* S. aureus* in throat, % (*n*/*N*)	36 (22/61)	37 (17/46)	45 (22/49)	20 (8/40)	35 (12/34)
* S. aureus* in perineum, % (*n*/*N*)	34 (21/61)	30 (14/46)	37 (18/49)	22 (9/41)	23 (8/35)
* S. aureus* in skin lesions, % (*n*/*N*)	48 (11/23)	31 (4/13)	50 (4/8)	33 (4/12)	33 (1/3)
* S. aureus* at any site, % (*n*/*N*)	62 (38/61)	63 (29/46)	61 (30/49)	46 (19/41)	49 (17/35)
* S. aureus* at ≥ 2 sites at the same occasion, % (*n*/*N*)	43 (26/61)	30 (14/46)	41 (20/49)	22 (9/41)	31 (11/35)
Infections					
Self-reported SSTIs, % (*n*/*N*)	44 (27/61)	43 (23/54)	35 (18/52)	33 (16/49)	22 (9/41)
Self-reported hospital admissions due to injection related infections, % (*n*/*N*)	7 (4/61)^a^	2 (1/54)^b^	0 (0/52)	0 (0/49)	0 (0/41)

^a^Sepsis (*n* = 1); sepsis; infection upper limb (*n* = 1); endocarditis (*n* = 1) and septic arthritis from *S. aureus* (*n* = 1).

^b^Septic arthritis (*n* = 1).

### *S. aureus* carriage

**Figure 1:**
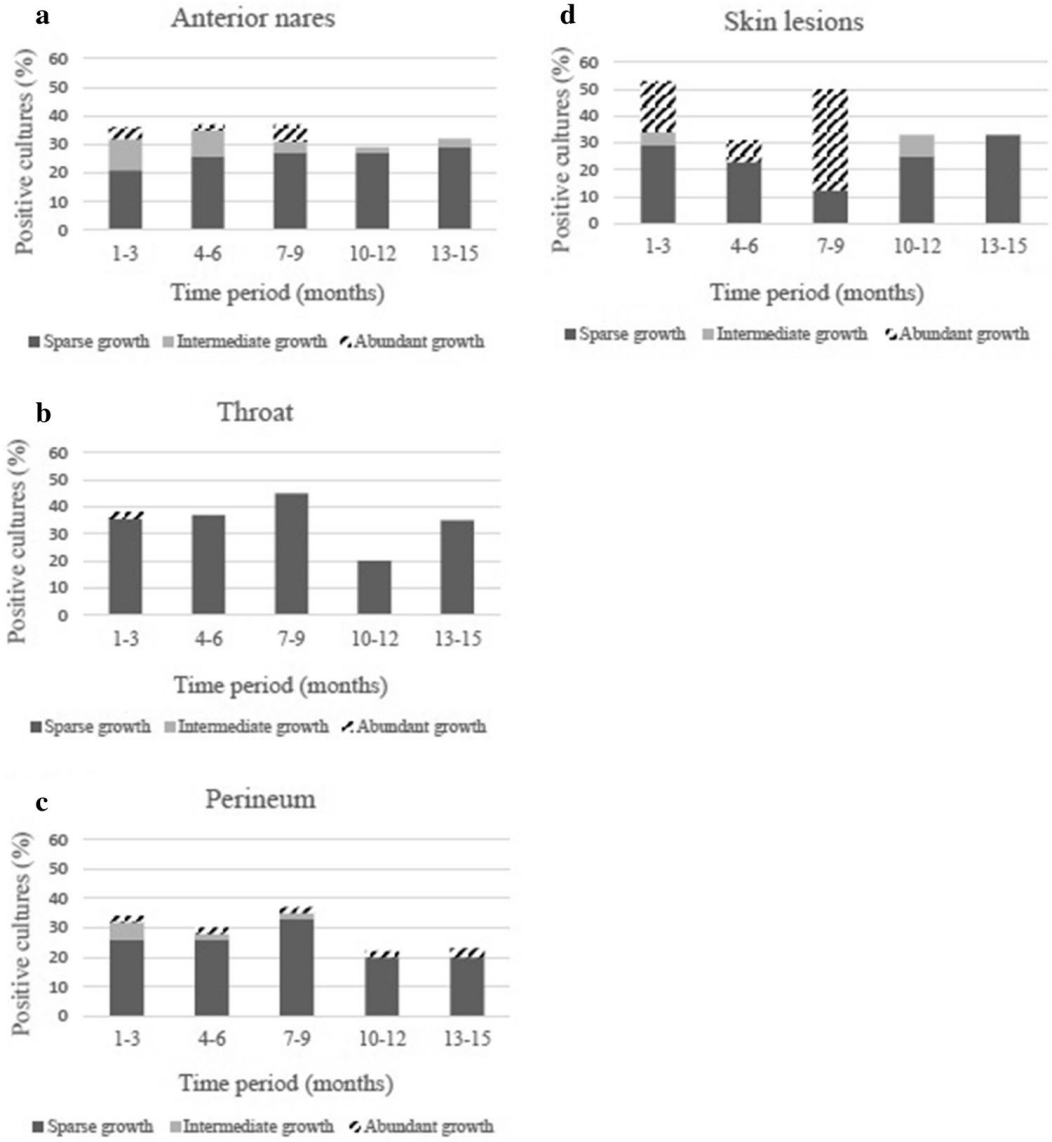
**(a)** Semi-quantification of *S. aureus* growth from anterior nares. Data are presented as the percentage of the total number of cultures analyzed during each 3-month period. (**b)** Semi-quantification of *S. aureus* growth from throat. Data are presented as the percentage of the total number of cultures analyzed during each 3-month period. (**c)** Semi-quantification of *S. aureus* growth from perineum. Data are presented as the percentage of the total number of cultures analyzed during each 3-month period. (**d)** Semi-quantification of *S. aureus* growth from skin lesions. Data are presented as the percentage of the total number of cultures analyzed during each 3-month period.

In total, 767 *S. aureus* cultures were performed during the 15-month period of data collection. Carriage with *S. aureus* in any body site during a three-month period ranged from 46% (month 10–12) to 63% (month 4–6). Depending on measuring point, the proportion with anterior nares carriage was 29–39%, throat carriage 20–45% and perineum carriage 22–37%. In skin lesions, *S. aureus* was detected in 31–50% of the cases (Table 2). The majority of cultures positive for *S. aureus* showed sparse growth, but in skin lesions abundant growth was also relatively common (Fig. 1a–d).

### Factors associated with self-reported SSTI

During the study period, 823 structured interviews were conducted. In total, 46/61 PWID (75%) reported any SSTI during this period. Self-reported SSTIs during a 3-month period were ranging from 22% (months 13–15) to 44% (months 1–3) (Table 2). In both univariate and multivariate analysis, self-reported SSTIs were associated with *S. aureus* carriage in perineum (OR 5.08 [95% CI 1.45–17.73] vs. OR 4.19 [95% CI 1.01–17.38]). The same applied for an unstable housing situation (OR 12.83 [95% CI 1.56–105.81] vs. OR 16.06 [95% CI 1.70–151.64]). In univariate analysis, an association was also found for *S. aureus* carriage in skin lesions (OR 1.48 [95% CI 1.21–1.81]). Multivariate analysis was not done for *S. aureus* carriage in skin lesions, since all of these participants reported at least one SSTI (Table 3).

**Table 3:** Factors associated with self-reported skin and soft tissue infections (SSTIs).

Total *N* = 61	No SSTI reported (*n* = 15)	SSTI reported (*n* = 46)	Univariate	Multivariate^c^
			OR (95% CI) *p-*value	OR (95% CI) *p*-value
Gender				
Male, *n* (%)	10 (67)	25 (54)	0.60 (0.18–2.02)^a^ 0.55	1.04 (0.23–4.72) 0.96
Median age (years ± SD) (range)	47 ± 10.60 (24–62)	40 ± 12.44 (18–65)	0.96 (0.91–1.01)^b^ 0.10	0.95 (0.89–1.01) 0.07
Housing situation unstable, *n* (%)	1 (7)	22 (48)	12.83 (1.56–105.81)^a^ 0.01	16.06 (1.70–151.64) 0.02
Shower access unstable, *n* (%)	0	10 (22)	1.28 (1.10–1.49)^a^ 0.06	N/A
*S. aureus* in perineum^d^, *n* (%)	5 (33)	33 (72)	5.08 (1.45–17.73)^a^ 0.01	4.19 (1.01–17.38) < 0.05
*S. aureus* in nares^d^, *n* (%)	7 (47)	28 (61)	1.78 (0.55–5.75)^a^ 0.38	N/A
*S. aureus* in throat^d^, *n* (%)	7 (47)	31 (67)	2.36 (0.72–7.74)^a^ 0.22	N/A
*S. aureus* in skin lesions^d^,* n* (%)	0	15 (33)	1.48 (1.21–1.81)^a^ 0.01	N/A

Univariate analysis was done by Fischer’s exact test or logistic regression analysis and multivariate logistic regression.

^a^Fischer’s exact test. A *p* value < 0.05 was considered as statistically significant.

^b^Logistic regression.

^c^Logistic regression. Included covariates: age, gender, housing situation and *S. aureus* in perineum.

^d^Positive *S. aureus* culture at any time during the study period.

## Discussion

This study describes longitudinal carriage data in a hard-to-reach population in need of improved health care. This is in contrast to many carriage studies in the field that have a cross sectional design^11–13^. We confirm that PWID are frequent carriers of *S. aureus* and describe a surprisingly high 15-month prevalence of self-reported SSTIs in our cohort. Instable housing situation and *S. aureus* skin carriage are factors that are associated with SSTIs, indicating that hygiene factors could play a role and may be a target for prevention.

Our result, with a *S. aureus* carriage rate of 46–63% among PWID, are in line with earlier studies showing carriage rates from 39 to 67% in this group^11–13^. Among the general population, previous longitudinal studies on *S. aureus* nasal carriage have demonstrated persistent carriage in approximately 20% of the population, while about 30% are intermittent carriers and about 50% non-carriers^19^. In our study, *S. aureus* nasal carriage was detected in 29–39% among PWID, which indicates that PWID are more frequent carriers than people in general. The anterior nares has been shown to be the most frequent carriage site for *S. aureus* in humans^20^. Towards the end of the observation period for each individual, there seems to be a decline of the *S. aureus* carriage prevalence in the study sample. However, our sample size is too small to allow statistical calculations regarding this. It is possible that the study itself made the study participants more aware of the different hygiene issues linked to, e.g., injection procedures. Another explanation could be that well-functioning PWID had higher retention in the study.

During the 15-month study period, 75% of the included PWID reported at least one SSTI. The prevalence is surprisingly high, even compared to life-time data from international studies, reporting a prevalence of 46–68%^5–10^. Other skin manifestations, besides from infections, may have been reported as SSTIs, for example non-infected wounds and eczema. Another explanation could be the short interval between the interviews. Consequently, the participants may have been more observant, also reporting mild self-limited SSTIs, that otherwise would have been forgotten after a while. Three-month prevalence of SSTI in our study varied from 22 to 44% depending on measuring period. Other studies, from both Europe and the U.S., describe a 12-month prevalence of community acquired SSTI of 29–52%^6,9,10,21^. One study from our area demonstrates a 30-day prevalence of 14%^10^, indicating that even shorter observation periods are enough to detect the high prevalence of SSTIs in this group.

The demonstrated association between *S. aureus* carriage and self-reported SSTIs is significant, but with chosen design we cannot state whether there is a causality or not. Having unstable housing situation correlates with higher prevalence of reported SSTIs in multivariate analysis, which has been demonstrated before^22^. This indicates that other factors also play a role in the pathogenesis of SSTIs among PWID, and all factors that aggravate the trauma and irritation of the skin and veins are presumably relevant. Interestingly, a quite recent study reported high level of *S. aureus* survival and contamination of drug preparation equipment used in the preparation of Hydromorphone controlled-release, one of the most common opioids used in the PWID population^23^. Adequate skin hygiene before injection has been shown to decrease the risk for infections^2,16^, and there are yet many factors that have not been well explored in this context. However, it is plausible to assume that factors affecting the immune system, for example co-morbidity and malnutrition, also play a role. An association between female gender and higher risk for SSTI has been found in previous studies^10,21,22,24^, but in a quite recent study by Wright et al*.* such an association could not be found^8^. Neither was this observed in our present study. However, our limited population size could make such an association hard to find.

The gradual loss to follow-up, notified during the observation period, is a limitation of the study, but was less extensive than expected. In most cases, the reason for ending study participation early was admission to treatment centers. It is reasonable to assume that this could cause a bias, with the most well-functioning PWID remaining until study end. Moreover, data regarding SSTI prevalence are solely based on self-reported information, not confirmed with clinical examination. Despite this disadvantage, most similar studies in the field are based on self-reported data, which facilitates comparisons. Morrison et al*.* has also shown, by comparing semi-structured interviews with subsequent physical examination, that self-reporting of SSTI is a reliable method to establish prevalence^25^. Besides from *S. aureus*, SSTIs can also be caused by other bacteria, such as streptococcal species, gram-negative bacteria, and anaerobes^2–4^. There are data showing that 41% of SSTIs in PWID may be polymicrobial^26^. This may also limit our reasoning in this work, but in this study we have chosen to focus on *S. aureus*, since it has been shown to be the most prevalent pathogen. This has recently been demonstrated by MacLeod et al. in a retrospective material analysing 558 PWID admissions^27^. Summanen et al*.* has also demonstrated that *S. aureus* is the most common cause of cutaneous and subcutaneous abscesses in patients, regardless of any history of intravenous drug use^4^. Furthermore, in our study diagnostics focused on *S. aureus* without any additional data on antimicrobial resistance. Since Sweden is a low prevalence country for methicillin-resistant *S. aureus* (MRSA), the frequency of MRSA should be low in our material^28^. This has also been confirmed in a previous study from the same area^13^. Multilocus sequence typing, SpA typing or, preferentially, whole genome sequence (WGS) typing of the *S. aureus* isolates in this material would be of great importance for epidemiologic understanding in this cohort. Questions like spreading of certain strains within the cohort, as well as presence of certain virulence factors, could then be addressed and discussed further.

The findings in this study imply a need for further research, including clinical interventions, to reduce the burden of *S. aureus* carriage and the risk for SSTIs and subsequent complications among PWID. There are different strategies for harm reduction among PWID. Needle exchange programs offer sterile injection equipment, as well as surveillance and outpatient healthcare. The main aim of providing sterile injection equipment is to prevent transmission of bloodborne viruses, but preventing reuse of injection equipment is also aimed at decreasing the risk of bacterial infections. However, there is a need for intervention studies focusing more on hygiene aspects and its significance.

## Conclusion

To summarize, about half of the included PWID were carriers of *S. aureus* and 75% reported at least one SSTI during the study period. Having unstable housing situation and skin carriage were both factors associated with SSTIs. Despite a considerable loss to follow-up, we believe that our study is important since it highlights a prevalent health issue in a vulnerable population. Our results and collected data via a well-functioning NEP may enable further studies with focus on infection risk and prevention in the PWID group.

## Data Availability

The data supporting the findings of this study are available from the corresponding author upon reasonable request.
